# Stress avulsion of the tibial tuberosity after tension band wiring of a patellar fracture: a case report

**DOI:** 10.1186/1757-1626-2-9357

**Published:** 2009-12-19

**Authors:** Michael T Hirschmann, Björn Wind, Christian Mauch, Gesa Ickler, Niklaus F Friederich

**Affiliations:** 1 Department of Orthopaedic Surgery and Traumatology, Kantonsspital Bruderholz, CH-4101 Bruderholz, Switzerland

## Abstract

**Introduction:**

To the best of our knowledge there is no other report of an elderly patient who was surgically treated for a patellar fracture with tension band wiring and who subsequently suffered from an avulsion fracture of the tibial tuberosity. The combination of a patellar fracture and avulsion of the patellar ligament has only been described as complication after bone-patellar tendon-bone anterior cruciate ligament reconstructions. However, due to demographic changes and more elderly patients treated this injury may become more frequent in future.

**Case presentation:**

We present the case of an 81 year old female who sustained an oblique patellar fracture after a direct contact injury of the left knee when falling on ice. Consequently the patellar fracture was openly reduced and stabilized with tension band wiring. The follow-up was uneventful till three months after surgery when the patient noticed a spontaneous avulsion fracture of the tibial tuberosity (Ogden type 3). The tibial tuberosity fragment was reattached with two non-resorbable sutures looped around two modified AO cortical 3.5 mm long neck screws. Intraoperatively multiple bone cysts were seen. Biopsies were not taken to prevent further fragmentation of the tibial tuberosity. The patient was followed up with anteroposterior and lateral full weight bearing radiographs and clinical assessment at 6, 12 weeks and 6 months after surgery. Recovery was completely pain free with full satisfaction.

**Conclusion:**

In conclusion in elderly patients with a patella fracture a possible associated but not obvious fracture of the tibial tuberosity should be ruled out and the postoperative rehabilitation protocol after tension band wiring of the patella might have to be individually adjusted to bone quality and course of the fracture.

## Introduction

To the best of our knowledge there is no other report of an elderly patient who was surgically treated for a patellar fracture with tension band wiring and who subsequently suffered from an avulsion fracture of the tibial tuberosity. The combination of a patellar fracture and avulsion of the patellar ligament has only been described as complication after bone-patellar tendon-bone anterior cruciate ligament reconstructions. However, due to demographic changes and more elderly patients treated this injury may become more frequent in future.

## Case presentation

**Figure 1:**
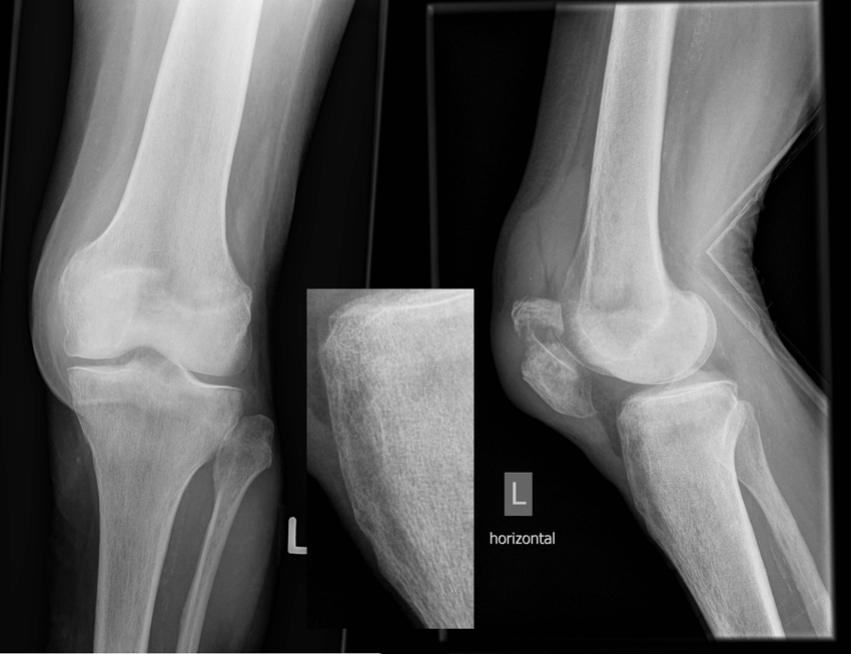
**Radiographs directly after fall on ice showing the displaced patellar fracture**. Anteroposterior and lateral radiographs showing the transverse displaced patellar fracture and magnified tibial tuberosity indicating no abnormality besides multi-lobular bone-cysts.

**Figure 2:**
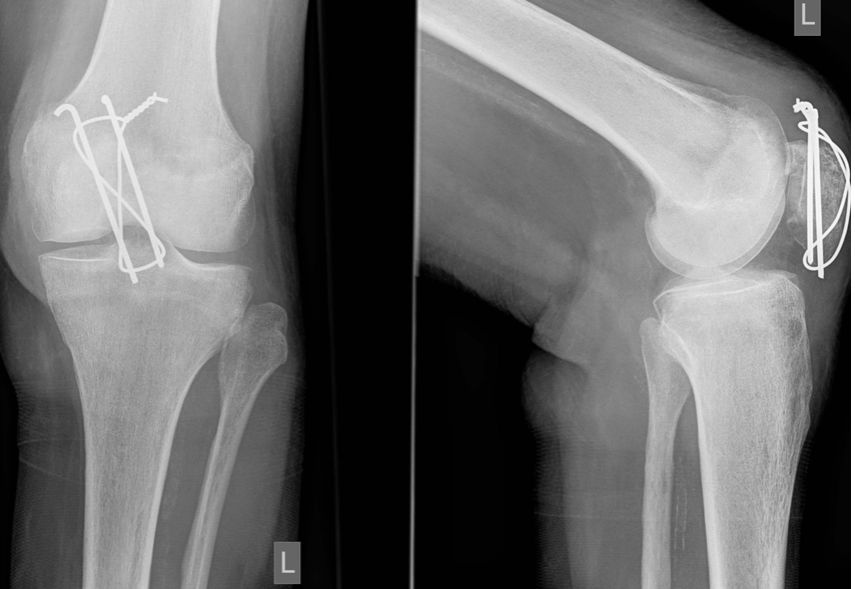
**Anteroposterior and lateral radiographs of left knee 3 months after patellar fracture and cerclage wiring (K-wires and wiring perpendicular to fracture line)**.

**Figure 3:**
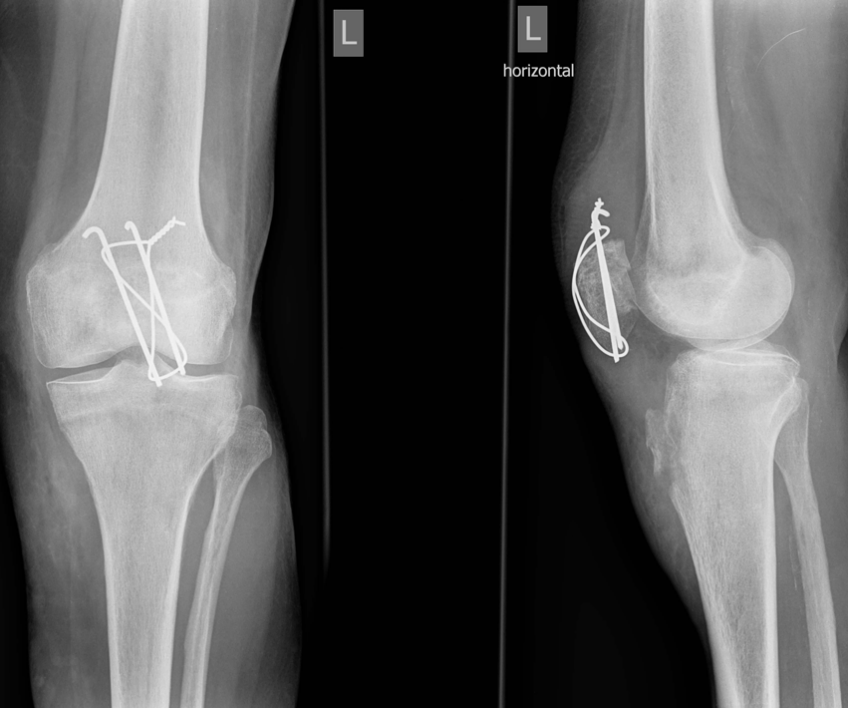
**Anteroposterior and lateral radiographs of left knee showing the avulsion fracture of the tibial tuberosity, a subtle proximalisation of the K-wires and intraarticular step due to a bony defect**.

**Figure 4:**
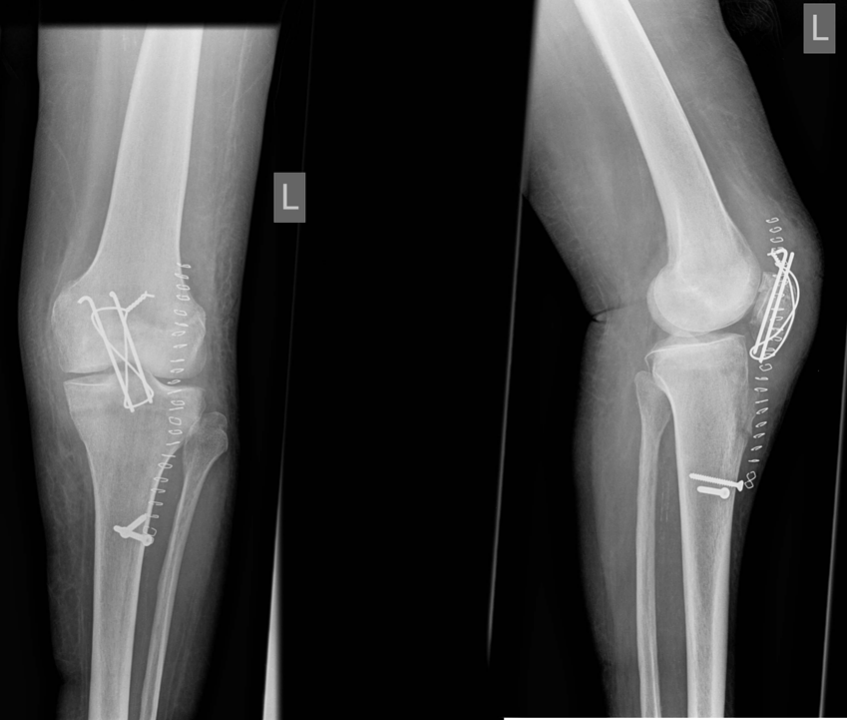
**Postoperative anteroposterior and lateral radiographs of left knee after refixation of avulsion fracture of the tibial tuberosity**.

**Figure 5:**
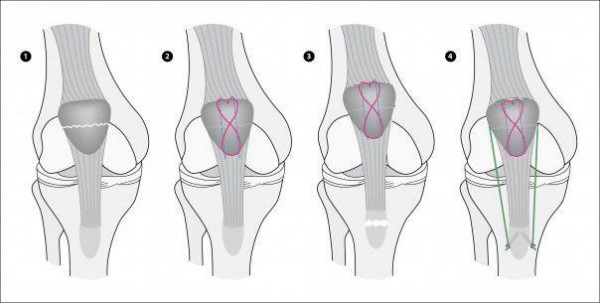
**Schematic drawing of the refixation technique of the tibial tuberosity with two AO cortical long neck screws and two FibreWire^®^- sutures**.

A 81 year old female had sustained a direct contact injury of the left knee due to a fall on ice. Radiologically a displaced oblique patellar fracture was proven in anteroposterior and lateral radiographs of the knee (fig. 1). No other injuries and/or fractures could be noted. At this time the patient was surgically treated with open reduction and tension band wiring of the patellar fracture (fig 2). Immediately after surgery full protected weight bearing was allowed for 6 weeks with an extension splint (SAMA, Salzmann Medico, St. Gallen, Switzerland). Anti-thrombotic prophylaxis was performed with low molecular weight heparin. Early functional physical therapy was initiated, for six weeks with limited passive flexion to 80°. Follow-up six weeks after surgery was done with clinical assessment and anteroposterior and lateral full weight bearing radiographs. At this time the activity related level of pain was rated as none. The patient was completely satisfied and passive range of motion for flexion/extension was 110°-0°-0°. Full weight bearing with unlimited range of motion was allowed after six weeks. Three and a half months after primary injury the patient felt a giving-way of the left knee when descending a stair during physiotherapeutic exercise. She heard a cracking sound and noted a stabbing pain in her lower leg. In anteroposterior and lateral radiographs an avulsion fracture of the tibial tuberosity (Ogden type 3a^1^) was observed (fig. 3). Retrospectively a multi-lobulated small cystic lesion at the tibial tuberosity was already present at the initial presentation. After a lateral parapatellar skin incision and medial parapatellar arthrotomy the tibial tuberosity fragment was refixed with two non-resorbable sutures (FibreWire^®^, Arthrex Swiss AG, Volketswil, Switzerland) which were looped around two modified 3.5 mm AO cortical long neck screws (fig. 4, 5). Intraoperatively multiple bone cysts were seen. Biopsies were not taken to prevent further fragmentation of the tibial tuberosity. Immediately after surgery protected full weight-bearing with crutches and an extension splint (SAMA, Salzmann Medico, St. Gallen, Switzerland) was allowed for two weeks. Anti-thrombotic prophylaxis with low molecular weight heparin was initiated. Passive flexion was initially limited to 35° for two weeks, then increased for the next two weeks to 70° and for the next two more weeks to full flexion under physiotherapeutic supervision.

**Figure 6:**
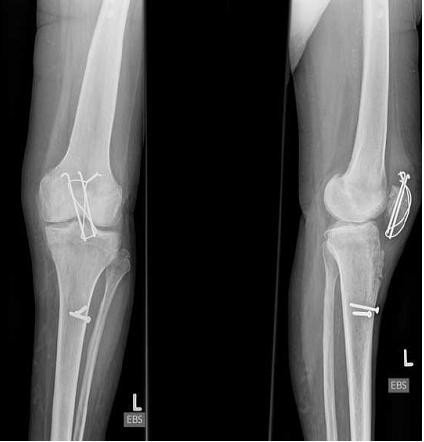
**Weight-bearing anteroposterior and lateral radiographs of left knee 6 months after refixation of the tibial tuberosity**.

The patient had a radiographic follow-up six weeks postoperatively with anteroposterior and lateral full weight bearing radiographs. Clinical assessment was done at 6, 12 weeks and 6 months after surgery. The patient was completely satisfied and pain free at the last follow-up. Range of motion was flexion/extension 125°-0°-0°. Radiographically the avulsion fracture had healed and there was no displacement of the tibial tuberosity (fig. 6).

## Discussion

Avulsion fractures of the tibial tuberosity are uncommon injuries [1-6]. In adolescents this type of fracture accounts for less than 3% of all epiphyseal plate injuries [6,7]. To our knowledge it has not been reported in the elderly. This case report is unique because it describes a case of a patient who was surgically treated for a patellar fracture with tension band wiring and who subsequently suffered from an avulsion fracture of the tibial tuberosity. The combination of a patellar fracture and avulsion of the patellar ligament has only been described as complication after bone-patellar tendon-bone anterior cruciate ligament reconstructions [8-10].

The aetiology of the tibial avulsion fracture in our case however remains unclear. It can only be speculated whether the fracture of the tibial tuberosity may be attributed to an overlooked injury of the tibial insertion of the patellar ligament at the time of the patellar fracture or whether the avulsion fracture may be a secondary stress fracture due to mechanical overloading. Even retrospectively there was no evidence of an avulsion to the tibial tuberosity, but a cystic lesion was apparent. Thus a secondary stress fracture seems to be a more reasonable cause of the injury. There were no other well known predisposing factors such as a patella infera, tight hamstrings, or a pre-existing Osgood-Schlatter's disease [7,11].

It might be the case that the course of the patellar fracture itself and the subsequent need for an oblique placement of the k-wires played a role in the development of the avulsion fracture of the tibial tuberosity. The distal k-wire may have altered the tension within the patellar ligament. The question however remains what was the cause of the cystic lesion at the tibial tuberosity. Most likely it was due to osteoporosis related decreased mechanical resistance of the tibial tuberosity [12].

One could argue that the early functional rehabilitation program have led to the avulsion injury and should have been modified with regards to the age-related inferior bone quality at the tibial tuberosity. The rehabilitation program was modified after the second surgery.

The goals of treatment of acute tibial tuberosity avulsion fractures are anatomic reduction of the fracture fragment, restoration of extensor mechanism alignment, and maintenance of congruency of the tibial articular surface. Non-operative treatment (closed reduction and cylinder cast immobilization) is only indicated in undisplaced Ogden type 1 and 2 fractures, which do mostly occur in children and adolescents [6,7]. In displaced avulsion fractures surgical treatment is indicated [1-7]. A variety of surgical methods such as transfixing pins or screws [6], staples [7], bone pegs [7], tension bands [6] or even direct suture [6] have been described for treatment of avulsion fractures of the tibial tuberosity. In our case a transfixation technique with a tension band wiring to neutralize the tension forces was performed. It led to a good functional result with a pain free patient at the last follow up control. No augmentation of bone, whether allo- or autograft, was necessary, but should be deliberately considered.

## Conclusion

In conclusion in elderly patients with a patella fracture an associated, not obvious fracture of the tibial tuberosity, should be ruled out. Postoperative rehabilitation protocol after tension band wiring should be individually adjusted to bone quality and course of the fracture.

## Consent

Written informed consent was obtained from the patient for publication of this case report and accompanying images. A copy of the written consent is available for review by the Editor-in-Chief of this journal.

## Competing interests

The authors declare that they have no competing interests.

## Authors' contributions

MTH reviewed the case and drafted the manuscript. BW participated in drafting the manuscript and case review. CM participated in drafting the manuscript and literature review. GI participated in drafting the manuscript and case review.

NFF participated in drafting the manuscript and case review. All authors read and approved the final manuscript.
